# Full-Arch Oral Rehabilitation in All-on-4 “M” Configuration Using Surgical Guides with Internal Cooling: A Clinical Case Report

**DOI:** 10.3390/jcm15031070

**Published:** 2026-01-29

**Authors:** Robert-Angelo Tuce, Monica Neagu, Vasile Pupăzan, Adrian Neagu, Stelian Arjoca

**Affiliations:** 1 Department of Functional Sciences, Victor Babes University of Medicine and Pharmacy Timisoara, 300041 Timisoara, Romania; tuce.robert@gmail.com (R.-A.T.); neagu.monica@umft.ro (M.N.); pupazan.vasile@umft.ro (V.P.); arjoca.stelian@umft.ro (S.A.); 2 Center for Modeling Biological Systems and Data Analysis, Victor Babes University of Medicine and Pharmacy Timisoara, 300041 Timisoara, Romania; 3 Department of Physics and Astronomy, University of Missouri, Columbia, MO 65211, USA

**Keywords:** dental implant bed preparation, thermal bone damage prevention, guided implant surgery, flapless guided surgery, custom surgical guides, internal irrigation

## Abstract

**Background/Objectives:** The All-on-4 technique is a minimally invasive approach for full-arch oral rehabilitation. In cases of anterior bone resorption, the classic All-on-4 configuration may be limited by insufficient bone for axial implant insertion. An effective alternative is the “M” configuration, where all four implants are inserted at approximately 30°. This report presents a clinical case of full-arch rehabilitation using personalized surgical guides with internal cooling, designed to optimize irrigation and prevent thermal bone damage. **Methods:** A 57-year-old female patient underwent digital planning and flapless guided implant surgery. At the maxilla, four DENTIS SQ implants were inserted in an “M”-shaped configuration (4 × 10 mm anterior, 4 × 14 mm posterior). Before insertion, implant beds were prepared using surgical templates with internal irrigation channels. At the mandible, four posterior implants (4 × 8 mm) were placed using dentally supported guides with internal cooling. The surgical guides were designed using Implastation and Blue Sky Plan, 3D-printed in biocompatible resin and sterilized before use. We performed the osteotomies under controlled irrigation with continuous saline flow through the integrated cooling channels. **Results:** The guides ensured accurate implant positioning. We did not observe intraoperative complications, and all implants achieved primary stability above 35 N·cm. Postoperative healing was uneventful, with minimal edema and no mucosal dehiscence. These indirect clinical indicators suggest that the guide also ensured effective cooling. Radiographic follow-up confirmed correct 3D positioning and intimate bone-implant contact. **Conclusions:** This case study shows that personalized surgical guides with integrated coolant channels may provide a safe and precise solution for flapless All-on-4 “M” rehabilitations, reducing thermal risks and enhancing surgical accuracy.

## 1. Introduction

Edentulism continues to represent a major challenge in oral rehabilitation, affecting masticatory function, esthetics, and patients’ quality of life [1]. The All-on-4 concept, introduced by Malo et al., has become one of the most predictable and efficient solutions for the complete restoration of edentulous arches [2]. It relies on the placement of four dental implants—two anterior axial and two posterior tilted—designed to avoid critical anatomical structures, such as the maxillary sinuses and the mandibular canal, while providing optimal biomechanical support for a full-arch fixed prosthesis [3,4].

Nevertheless, in patients with severe anterior alveolar ridge resorption, the classical All-on-4 configuration may lack adequate bone support for the axial insertion of the anterior implants. A viable alternative is the All-on-4 “M” configuration, whereby all four implants are inserted at an inclination of about 30°—two tilted mesially in the anterior region and two tilted distally in the posterior region—resulting in an “M”-shaped geometry. This configuration allows the use of longer implants within the available bone, avoiding penetration of the nasal cavity or maxillary sinus, while maintaining a favorable distribution of occlusal loads [3,4].

From a biomechanical standpoint, the “M” configuration offers improved anteroposterior spread and higher primary stability in atrophic maxillae, reducing the need for bone grafting or sinus lift procedures. Consequently, the treatment time and postoperative morbidity are significantly reduced [3,4].

Although the All-on-4 technique is well established, performing it in the “M” configuration remains surgically demanding. The accurate insertion of four tilted implants requires clinical experience and an excellent three-dimensional (3D) understanding of the patient’s anatomy. Small deviations in the drilling trajectory or implant angulation can compromise both the prosthetic alignment and biomechanical load distribution.

Guided surgery has therefore become an invaluable aid, translating virtual planning into highly accurate implant bed preparation, boosting the predictability of complex implant procedures [5,6,7].

However, during osteotomy, thermal control remains a critical factor for implant osseointegration. When the bone temperature exceeds 47 °C, even for short periods of time of the order of minutes, irreversible osteonecrosis may occur, jeopardizing the stability and long-term success of the implants [8,9,10,11].

Conventional surgical guides cover the target tissues and block the access of the cooling fluid to the drilling site, increasing the risk of excessive heat generation [12,13]. Surgical guides outfitted with internal irrigation channels effectively address this limitation [14,15,16,17]. By directing the saline flow precisely toward the drill tip, these guides combine geometric accuracy with optimal intraosseous temperature regulation, enhancing both surgical safety and predictability.

Surgical guides with internal coolant ducts have already been used in clinical practice. Alevizakos et al. designed an internally cooled surgical guide for an edentulous patient who needed two implants to ensure sufficient retention for her full denture [18]. They demonstrated the clinical applicability of their design without affecting the guide’s stability and accuracy of implant insertion. Orgev et al. proposed a simple design of a tooth-supported osteotomy guide, in which a properly placed fixation pin access channel served as an irrigation duct [19]. Teich et al. reported in vitro results proving that internally routed irrigation reduced the temperature elevation associated with guided osteotomy [10]. Moreover, they created an internally cooled osteotomy guide for a patient and used it in flapped surgery. Uzunov et al. proposed a dual-channel design for internally cooled osteotomy guides and demonstrated its intraoperative use [20]. Nevertheless, none of the above papers presented the entire therapeutic process, from digital planning to implant insertion and post-operative follow-up. This literature gap motivated the present study.

This case report presents a clinical case of full-arch oral rehabilitation using the All-on-4 “M” configuration combined with personalized, 3D-printed surgical guides featuring internal coolant channels. Moreover, it describes the integrated workflow—from cone beam computed tomography (CBCT) and intraoral scanning to virtual planning, guide fabrication, flapless guided surgery, implant insertion, provisional prosthetic restoration, and patient monitoring—emphasizing its advantages in terms of surgical precision, temperature control, and patient comfort. The main contribution of this work is the use of customized surgical guides with internal irrigation in a complex clinical context.

## 2. Case Presentation

### 2.1. Patient Data and Diagnosis

A 57-year-old female patient, without systemic pathologies, presented for full-mouth oral rehabilitation. Written informed consent was obtained from the patient for the clinical procedures and the publication of the anonymized results. This study was conducted in accordance with the ethical principles stated in the Declaration of Helsinki. The study protocol was approved by the Committee of Scientific Research Ethics of the “Victor Babes” University of Medicine and Pharmacy Timisoara (protocol No. 63/11.11.2025).

Clinical and radiological examination of the patient revealed total edentulism of the maxilla, restored with a complete removable denture, and bilateral posterior edentulism of the mandible (regions 45–48 and 35–38).

The clinical objective was to re-establish both masticatory function and esthetics. The selected treatment plan consisted of a full-arch maxillary rehabilitation using the All-on-4 “M” configuration of four individual implants.

### 2.2. Digital Diagnostic and Planning Workflow

**Figure 1:**
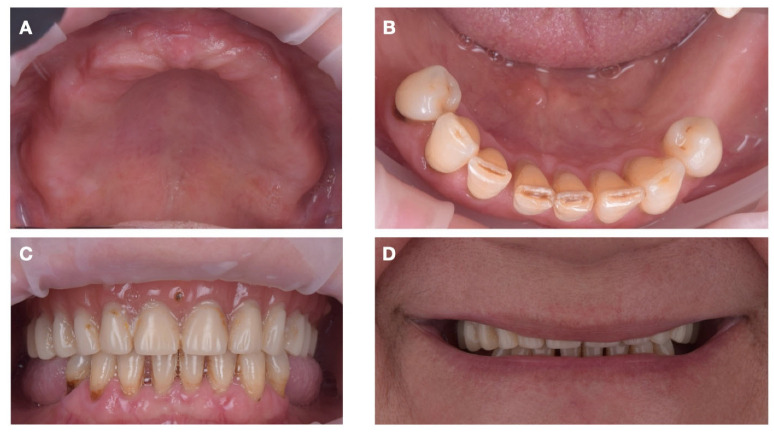
Intraoral photographs of (**A**) the maxillary arch, (**B**) the mandibular arch, and (**C**) the intermaxillary relationships; (**D**) exo-oral photograph of the smile.

A comprehensive digital diagnostic protocol was devised to correlate the patient’s anatomic conditions with prosthetic and biomechanical requirements. The clinical analysis included the evaluation of the patient’s facial profile, lip support, smile line (Figure 1D), and occlusal relationships (Figure 1A–C). The edentulous mucosa was inspected for thickness, elasticity, and stability of the existing denture.

**Figure 2:**
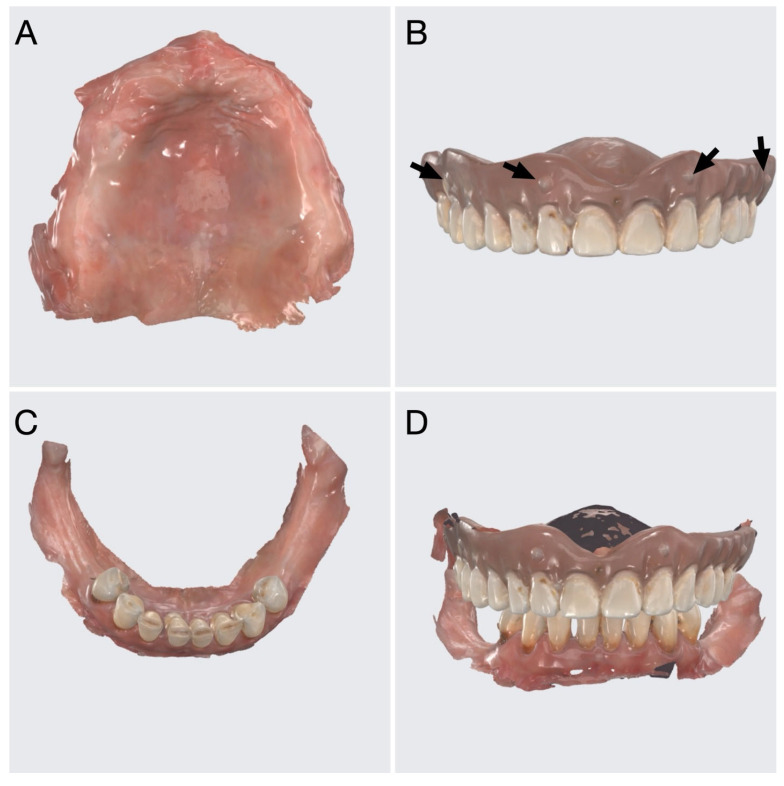
Intraoral scans of (**A**) the maxillary edentulous field, (**B**) the complete denture with radiopaque markers (arrows), (**C**) the mandibular arch, and (**D**) the intermaxillary relationship.

To align radiological and prosthetic data, before CBCT acquisition, radiopaque markers made of composite resin were placed on the vestibular surface of the maxillary denture (Figure 2B). Subsequently, intraoral scans of the edentulous maxilla (Figure 2A), the denture with markers (Figure 2B), the mandibular arch (Figure 2C), and the intermaxillary relationship (Figure 2D) were obtained using a Medit I700 scanner (Medit, Seoul, Republic of Korea). The scan of the denture was saved in STL file format.

**Figure 3:**
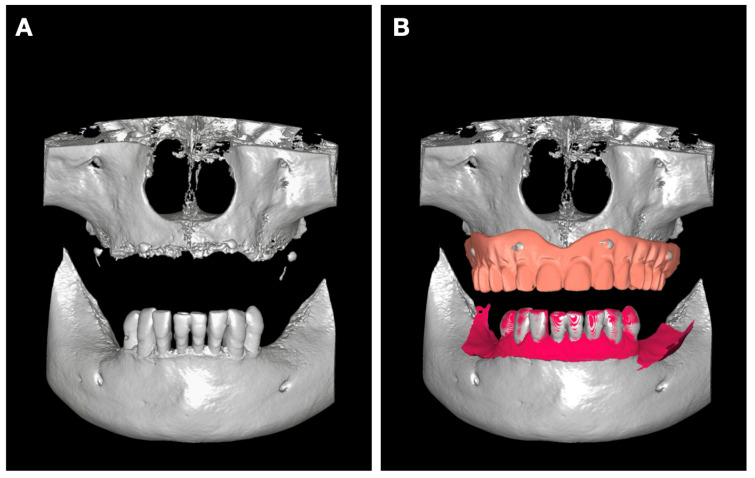
(**A**) 3D reconstruction from CBCT; (**B**) overlay of intraoral scans on the CBCT reconstruction. The maxillary prosthesis was overlaid using radiopaque markers, while the lower arch scan was aligned using dental units as references.

For a precise 3D assessment, a full CBCT scan was performed (Figure 3A). The images were analyzed for alveolar ridge morphology, sinus dimensions, mandibular canal position, and available bone height and width.

The STL files were aligned with CBCT data through radiopaque marker registration using the Implastation software (ProDigiDent, Batavia, IL, USA) (Figure 3B). We first aligned the markers and then verified, in both axial view and cross-section, that the contour of the prosthesis acquired by optical scanning matches that provided by CBCT.

**Figure 4:**
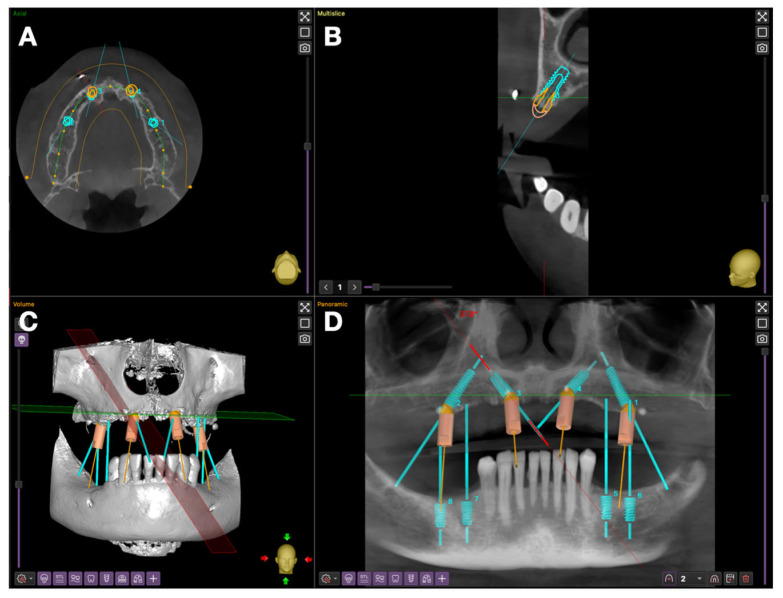
Dental implant planning: (**A**) axial section of the maxilla; (**B**) section of implant 12 showing its subcrestal positioning and the presence of a radiopaque marker; (**C**) 3D reconstruction; (**D**) panoramic view illustrating the implant positions and the “M” configuration of the maxillary implants.

A multiplanar analysis performed in Implastation revealed moderate anterior ridge resorption and accentuated posterior atrophy. Therefore, an All-on-4 “M” configuration was chosen, in which anterior implants were tilted mesially and posterior implants distally to avoid the maxillary sinuses (Figure 4).

The treatment plan for the maxilla involved the placement of four DENTIS SQ implants (Dentis, Daegu, Republic of Korea):

- 4 × 10 mm in regions 12 and 22 (anterior, tilted mesially);
- 4 × 14 mm in regions 16 and 26 (posterior, tilted distally).

Each implant was equipped with a 30° multi-unit abutment (MUA) with 1.5 mm gingival height, allowing parallelization and an FP1-type prosthetic design.

At the mandible, four DENTIS SQ implants (4 × 8 mm) were planned in regions 35–36 and 45–46, using a dentally supported guide based on the existing crowns for 3D stabilization (Figure 4D).

To enhance the cooling performance, internal irrigation channels were simulated digitally in Blue Sky Plan Version V5.06. (Blue Sky Bio, Libertyville, IL, USA), each represented by a 2 mm × 8 mm virtual canal oriented toward the bone-drill contact point.

This digital workflow enabled accurate 3D alignment, avoidance of anatomic risks, and integration of irrigation channels directly into the surgical guide design.

### 2.3. Surgical Guide Design and Fabrication

**Figure 5:**
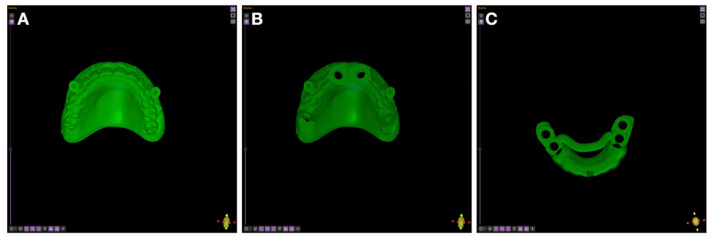
Design of surgical guides without internal cooling channels: (**A**) guide for drilling the positioning pin locations of the maxillary guide; (**B**) maxillary surgical guide; (**C**) mandibular surgical guide.

After completing the implant planning and fixation pins positioning in Implastation Version 5.3.2. (ProDigiDent, Batavia, IL, USA), the digital files were exported in STL format. For the maxilla, two separate guides were created: (i) a pin-drilling stabilization guide duplicated from the maxillary denture and positioned in perfect occlusion (Figure 5A), and (ii) the main guide for implant insertion (Figure 5B). For the mandible, a single, tooth-supported, sleeveless guide was designed (Figure 5C).

**Figure 6:**
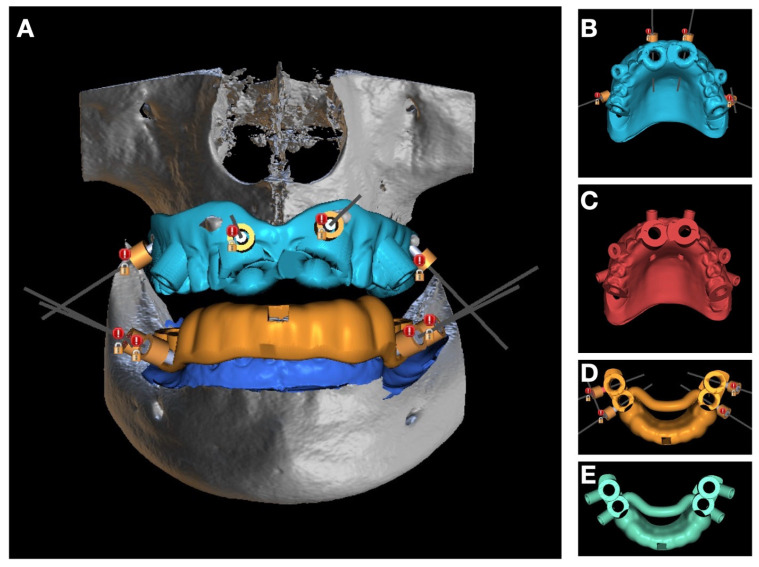
(**A**,**B**,**D**) planning of the 8 mini-implants for the creation of internal cooling sleeves; (**C**,**E**) digital designs of the osteotomy guides featuring internal cooling channels for each implant position.

To integrate cooling ducts, the STL files were imported into Blue Sky Plan V5.06. (Blue Sky Bio, Libertyville, IL, USA). For each planned implant, a digitally defined irrigation channel was added as a 2 mm-diameter, 8 mm-long virtual mini-implant, oriented precisely toward the drill–cortical contact point (Figure 6A,B,D). These channels were dimensioned with a 3.5 mm guiding sleeve diameter, 3.5 mm length, offset of 0 to ensure a steady, laminar saline flow to the active osteotomy site. Positions were adjusted to avoid anatomic conflicts and to maintain an optimal route between the physiodispenser tubing and the working area. Using the “scan appliance” workflow, the classical sleeveless guides were converted into internally cooled guides (Figure 6C,E). A volumetric integrity check was performed to prevent interference between irrigation ducts and drill sleeves and to optimize wall thickness for mechanical stability.

The finalized models were prepared in Accuware V3.2.1.109 (Shining 3D, Hangzhou, China) and printed on an AccuFab-L4D 3D printer (Shining 3D, Hangzhou, China) using SG01 biocompatible resin (Shining 3D, Hangzhou, China), targeting ±100 µm size accuracy (layer height 100 µm).

Post-processing included two-stage cleaning with isopropyl alcohol (Kynita, Budești, Romania), drying, and final UV-curing per manufacturer specifications. Channel transparency and permeability were verified, and compatibility with physiodispenser tubing was confirmed. The guides were then sterilized in an autoclave at 121 °C for 15 min. After the thermal treatment, no deformation or dimensional change in the guides was observed.

**Figure 7:**
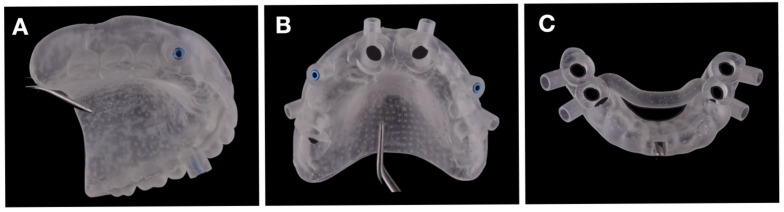
Three-dimensional-printed surgical templates used in this study: (**A**) guide for drilling the holes for the insertion of fixation pins; (**B**) maxillary surgical guide; (**C**) mandibular surgical guide.

Figure 7 shows photographs of the 3D-printed surgical templates. To assess clinical readiness, we test-fitted all guides on printed jaw models to confirm passive seating and occlusal stability. Pre-operative bench tests documented a passive fit within <100 µm, allowing safe transition to surgery.

The maxillary workflow utilized two guides (a fixation pin insertion guide and an implant site preparation guide with internal cooling), whereas the mandibular workflow employed a single, tooth-supported implant bed osteotomy guide with internal cooling (Figure 7A–C).

### 2.4. Surgical Protocol

The surgical intervention was carried out in two distinct stages corresponding to the maxillary and mandibular arches, both performed under guided, minimally invasive, flapless conditions.

Immediately before surgery, the patient was rinsed with 0.2% chlorhexidine solution, and perioral skin disinfection was performed before draping the sterile field. We performed all procedures under aseptic conditions, using sterile instrumentation and a physiodispenser with controlled irrigation. We carefully monitored coolant flow by visual inspection, but did not measure temperature elevations caused by surgical drillings.

#### 2.4.1. Stage I—Maxillary Rehabilitation Using the All-on-4 “M” Configuration

The first surgical stage involved the maxilla, rehabilitated through the All-on-4 “M” technique. Local anesthesia was administered by infiltration with articaine 4% containing epinephrine 1:100,000, and the flapless procedure was performed, preserving the periosteal vascular supply.

**Figure 8:**
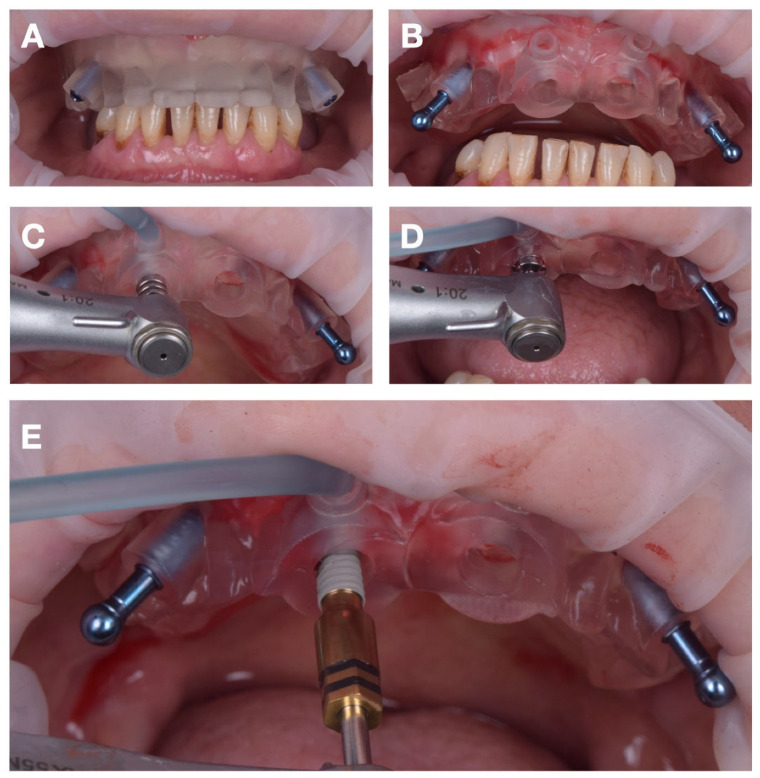
Surgical sequence for maxillary implant insertion: (**A**) drilling the pin holes with the stabilization guide in occlusion; (**B**) fixing the maxillary surgical guide in position with pins; (**C**) cutting the mucoperiosteum using the mucotome; (**D**) guided drilling; (**E**) implant insertion.

First, the fixation pin insertion guide—replicating the maxillary denture base—was positioned in occlusion and centered along the midline (Figure 8A). Once its stability was confirmed, two holes were drilled through the pin guides at 300 rotations per minute (rpm) under continuous irrigation.

After preparing the fixation sites, the stabilization guide was removed and replaced with the main surgical guide (Figure 8B). The guide was anchored using two fixation pins (Neodent, Curitiba, Brazil), providing rigid three-dimensional stability throughout the subsequent drillings.

The physiodispenser irrigation tubing was connected to the guide’s lateral input port, directing saline solution at approximately 23 °C through the internal channels toward the osteotomy site. This setup ensured continuous laminar cooling during each drilling sequence. Osteotomies were performed in accordance with the manufacturer’s protocol for DENTIS SQ implants, and the four implants were inserted according to the digital plan (Figure 8C–E).

The primary stability of all implants exceeded 35 N·cm, allowing immediate placement of multi-unit abutments (MUA). The procedure was completed without flap elevation, ensuring minimal bleeding and optimal preservation of soft tissues.

Immediately after implant placement, scan bodies were attached to the MUAs, and a digital intraoral scan was performed using a Medit i700 scanner (Medit, Seoul, Republic of Korea) to capture the 3D position of the implants. The STL files were transferred to the laboratory for computer-aided design/computer-aided manufacturing (CAD/CAM) design and fabrication of a screw-retained polymethyl methacrylate (PMMA) provisional restoration.

#### 2.4.2. Stage II—Mandibular Posterior Implant Placement and Fixation of the Maxillary Provisional Restoration

The second session began with the fixation of the maxillary screw-retained provisional prosthesis, which had been previously designed and fabricated based on the digital impression obtained at the end of Stage I. The PMMA restoration was verified on the printed model and intraorally seated on the MUAs. Passive fit, screw access, and occlusion were carefully checked before final tightening. The provisional prosthesis was fixed using prosthetic screws torqued to 15 N·cm, ensuring stable and immediate esthetic rehabilitation of the maxillary arch.

**Figure 9:**
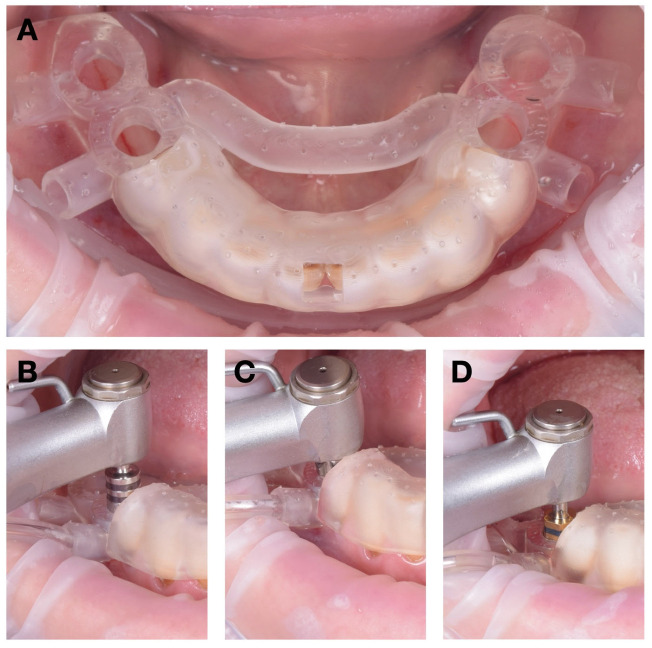
Surgical sequence for mandible implant insertion: (**A**) positioning the surgical guide over existing crowns; (**B**) cutting of the mucoperiosteum using the mucotome; (**C**) guided drilling; (**D**) implant insertion.

We next proceeded to the posterior mandibular implant placement. A tooth-supported internally cooled guide was used (Figure 9A), stabilized by occlusal contact with the remaining dentition. This design allowed accurate transfer of the digital plan while maintaining full visibility and irrigation control during surgery.

Four implants were inserted in positions 35–36 and 45–46 in accordance with the initial plan. All osteotomies were performed under continuous internal irrigation through the integrated channels, which maintained a constant saline flow at 23 °C to the drilling site (Figure 9B,C).

After implant placement, primary stability values exceeded 35 N·cm, confirming optimal bone quality and torque conditions for healing screws (Figure 9D).

## 3. Results

### 3.1. Primary Stability and Thermal Control

All implants achieved excellent primary stability. The insertion torque values ranged from 35 to 45 N·cm for both arches, indicating dense bone contact and optimal mechanical engagement.

Immediately after placement, Implant Stability Quotient (ISQ) values were measured using resonance frequency analysis. For the maxillary implants, ISQ readings were consistently above 70, validating the conditions for immediate loading with the screw-retained provisional restoration fabricated between stages. At the mandibular level, ISQ values also exceeded 80, confirming high initial stability and allowing the immediate placement of healing abutments without risk of micromovement or impaired osseointegration.

During all osteotomies, the saline coolant, maintained at about 23 °C, was directed precisely toward the drill–bone interface, ensuring efficient laminar flow throughout the drilling trajectory. Visual inspection through the transparent resin confirmed continuous coolant flow and the absence of coolant stagnation or reflux.

No intraoperative signs of overheating were observed, such as bone odor, smoke, or tissue whitening.

### 3.2. Accuracy of Implant Placement

The accuracy of implant positioning was verified through visual comparison between the digital planning created in Implastation and the postoperative intraoral scans obtained after guided implant placement.

**Figure 10:**
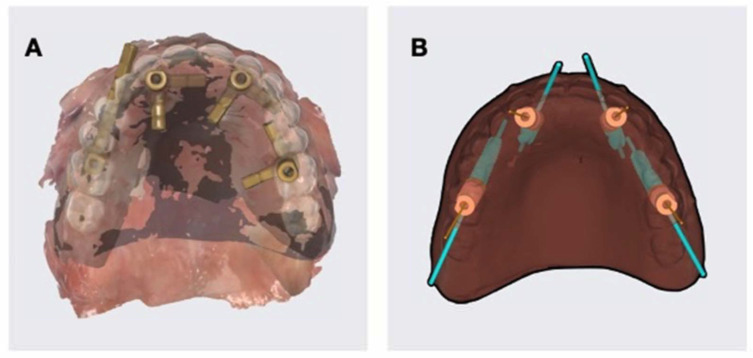
(**A**) Overlay of the initial prosthesis and edentulous field scans onto the dental implant position scan; (**B**) implant planning in Implastation, including implants, multi-unit abutments, and scan bodies.

Immediately after surgery, scan bodies were mounted on the MUAs, and an intraoral scan was performed to capture the definitive three-dimensional position of the implants. This scan was then superimposed with the scan of the patient’s initial complete denture, creating a single digital model that accurately represented the postoperative prosthetic situation (Figure 10A).

In parallel, the initial virtual plan created in Implastation (Figure 10B) displayed the pre-planned implant positions together with the corresponding MUAs, scan bodies, and the prosthetic setup designed from the initial denture.

Comparison of the postoperative superimposed scan (Figure 10A) with the preoperative digital simulation (Figure 10B) revealed a clear positional correspondence between the planned and the actual implant trajectories. The implant emergence profiles aligned with the intended prosthetic axes, confirming that the guided osteotomy protocol accurately transferred the digital plan to the clinical setting.

**Figure 11:**
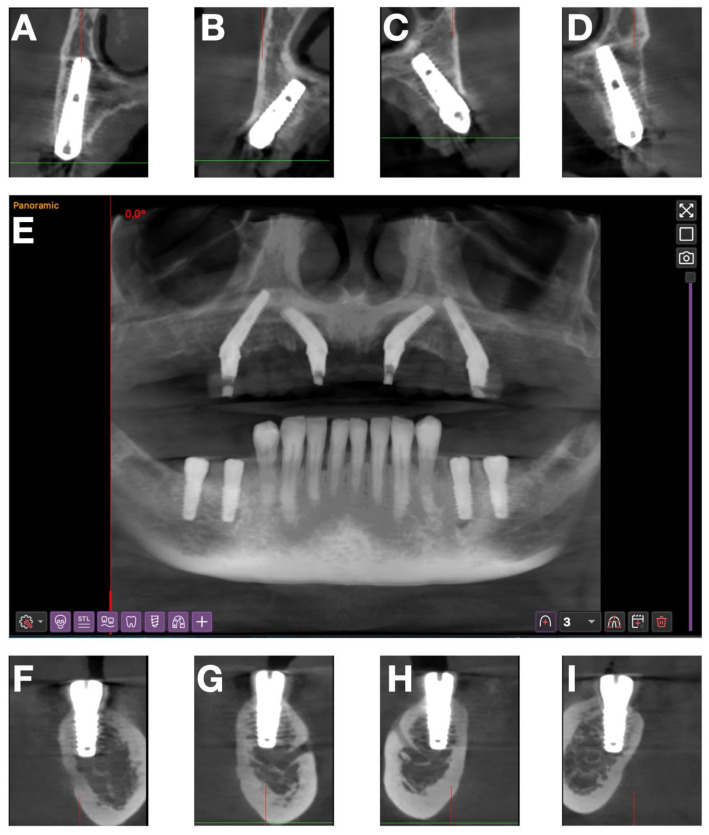
Postoperative CBCT: (**A**–**D**) sections at the level of maxillary implants 16, 12, 22, 26; (**E**) panoramic view; (**F**–**I**) sections at the level of mandibular implants 46, 45, 35, 36.

These qualitative observations were corroborated with the postoperative CBCT evaluation (Figure 11), which showed correct 3D alignment, parallelism of the anterior and posterior implants, and anatomic safety zones.

These results demonstrate that personalized surgical guides with integrated coolant channels can maintain the same level of geometric precision as standard surgical guides.

### 3.3. Postoperative Evolution and Patient Feedback

The postoperative evolution was clinically favorable and complication-free. Both arches healed without mucosal dehiscence, infection, or signs of inflammation. In the immediate postoperative period, the patient presented no edema and minimal discomfort, which subsided within the first 48 h under standard analgesic and anti-inflammatory medication. No hematoma or neurosensory alterations were reported.

**Figure 12:**
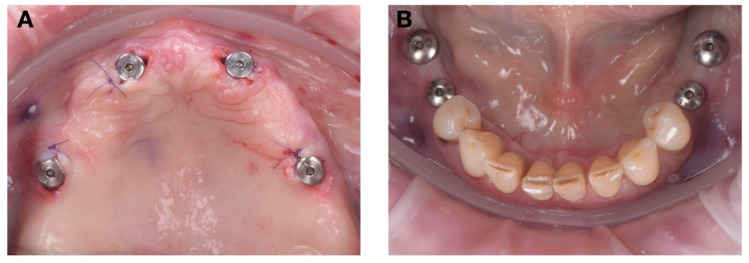
Postoperative photographs immediately after the procedure: (**A**) maxillary region; (**B**) mandibular region.

The flapless surgical approach, combined with the use of internally cooled guides, contributed to the minimal postoperative trauma observed. The continuous irrigation during osteotomy reduced bone heating and soft tissue dehydration, contributing to the fast recovery and stable healing of the peri-implant mucosa (Figure 12).

At the 7-day follow-up, the soft tissues appeared healthy, with normal color and contour. The absence of inflammation indicated optimal primary closure of the mucosa around the healing abutments.

At one month post-surgery, the peri-implant tissues were completely epithelialized, and the patient reported full comfort during mastication and speech.

Overall, the guided surgery ensured not only accurate implant positioning but also favorable postoperative outcomes—reduced swelling, fast healing, and immediate functional rehabilitation—resulting in self-reported patient satisfaction.

### 3.4. Prosthetic Restoration

**Figure 13:**
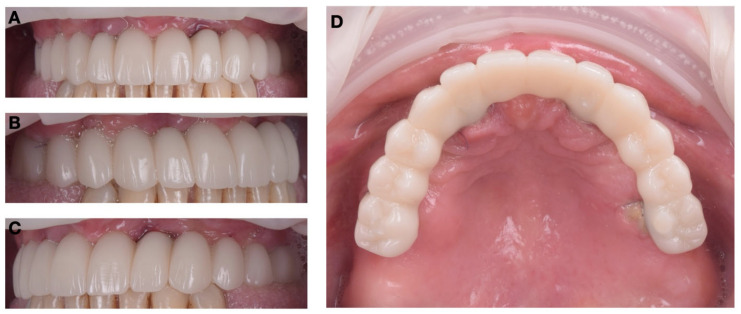
Photographs of the provisional prosthetic restoration of the maxillary arch: (**A**) frontal norm; (**B**) right lateral norm; (**C**) left lateral norm; (**D**) occlusal norm.

Following the completion of both surgical stages and confirmation of implant stability, the maxillary screw-retained provisional prosthesis was finalized and installed as planned (Figure 13A–D).

The design of the restoration was based on the intraoral digital scan performed after the first surgical stage, ensuring an accurate transfer of the implant positions and emergence profiles into the CAD environment. Using CAD/CAM technology, the provisional prosthesis was digitally designed and milled from high-density PMMA to ensure optimal mechanical strength and esthetic translucency.

Before delivery, the restoration was verified on the printed model to ensure passive fit and correct screw alignment. The intraoral installation confirmed precise seating on all MUAs without tension or occlusal interference. Final fixation was achieved using prosthetic screws torqued to 15 N·cm, followed by sealing of the access channels with composite resin.

The occlusal adjustments were minimal, confirming the accuracy of the digital planning and guided implementation. The result displayed a stable occlusal plane, balanced contacts, and satisfactory phonetic and esthetic outcomes. The gingival contours appeared natural, and the transition line was concealed within the smile zone, resulting in an excellent integration with the patient’s facial esthetics.

At the one-month follow-up, the restoration remained functionally stable, with no screw loosening, resin fracture, or mucosal irritation. The patient reported complete satisfaction with comfort, function, and esthetic appearance, appreciating the immediate return to a fixed dentition and the reduced treatment time.

Overall, the guided surgery combined with a fully digital prosthetic workflow enabled the predictable realization of an implant-supported fixed rehabilitation that met clinical success criteria.

## 4. Discussion

Thermal control during implant bed osteotomy remains one of the key determinants of osseointegration and long-term success. Experimental studies have established that whenever the bone temperature exceeds 47 °C for more than one minute, irreversible necrosis of the osteocytes occurs, leading to impaired primary stability and delayed integration [12]. In guided surgery, this risk can be exacerbated by the limited access of coolant to the drilling site due to the guide’s intimate contact with the mucosa or bone surface [8].

According to previous in vitro studies, internal irrigation channels integrated into 3D-printed guides represent an effective solution to mitigate heat accumulation during drilling. Several studies confirmed that guides with internal irrigation channels maintain bone temperature 4–6 °C lower than conventional surgical guides, significantly reducing the potential for thermal bone injury [10,21,22]. Traditional external irrigation often fails to reach deeper drilling sites, particularly under thick mucosa or dense cortical bone, resulting in localized temperature spikes and potential bone overheating [8,13]. By contrast, internal irrigation channels deliver the coolant directly to the critical zone, improving both thermal efficiency and surgical visibility. In vitro experiments demonstrated that the integration of internal irrigation channels into 3D-printed surgical guides lowered the maximum drilling temperature by more than 25% compared with standard guides without internal cooling [13]. Subsequently, a follow-up study confirmed these findings on standardized alveolar bone models, showing a significant reduction in peak temperature and faster thermal recovery when using guides with internal irrigation channels compared to conventional templates [16].

In the present clinical application, the internal coolant channels enabled laminar saline flow directly to the drill-bone interface, as confirmed by visual inspection during implant bed preparation. The favorable thermal conditions translated into stable implant insertion torque, high ISQ values (>70), and the possibility of immediate maxillary loading without compromising tissue health. These cumulative results support the biological and operational benefits of internally cooled guide designs in preserving bone vitality and ensuring safe and predictable implant placement. Although we did not measure intraosseous temperatures during implant bed preparation, the above in vitro works and the satisfactory clinical outcomes recorded in this case study suggest that intraoperative temperatures remained in the safe zone throughout all osteotomies.

Guided surgery provides remarkable accuracy in transferring the digital plan to the operative field. Qualitative evidence regarding the excellent correlation between the postoperative intraoral scans and the virtual plan confirmed the high accuracy of the proposed guided implantology protocol. This observation is consistent with the results of recent quantitative investigations. A systematic review of clinical studies found mean global deviations of around 0.65 mm coronally, 1.10 mm apically, and ~3.9° angularly, clearly favoring computer-assisted workflows over freehand implant placement [23]. A randomized clinical trial on immediate placement likewise reported much lower angular deviation for guided surgery (≈0.8°) versus freehand (≈6.1°), with smaller linear errors at entry and apex for the guided group [6]. Additional comparative work confirms that fully guided approaches outperform pilot-guided and freehand for immediately placed implants, reducing global platform/apex deviations and improving prosthetic predictability [7].

The correspondence we observed between the preoperative simulation in Implastation and the postoperative scan-denture overlay, along with a CBCT verification, fits this body of evidence and indicates that adding internal irrigation channels did not compromise the mechanical stability or dimensional accuracy of the guides. This finding is in agreement with the results reported by Alevizakos et al. [18].

Another essential aspect demonstrated by this case study is related to the flapless approach, which preserves the periosteal blood supply, minimizes postoperative edema, and accelerates mucosal healing. Recent comparative and meta-analytic studies have confirmed that flapless implant surgery results in less marginal bone resorption, reduced postoperative discomfort, and faster recovery compared with traditional flap elevation techniques [24]. Similarly, a prospective comparative study on immediate loading protocols reported faster soft tissue healing and reduced postoperative morbidity in patients treated with flapless guided implant placement [25]. Indeed, immediate loading was found to be a reliable option for implant-supported fixed partial prostheses across various protocols [26].

In the present case study, limited postoperative discomfort and rapid tissue epithelialization confirmed the biological benefits of a minimally invasive approach based on surgical templates with internally routed irrigation. Preserving keratinized mucosa and avoiding periosteal detachment further contributed to long-term soft tissue stability and esthetic outcomes.

When comparing this approach with other full-arch rehabilitation strategies, such as classical All-on-4 or zygomatic-anchored concepts, the “M” configuration offers an appealing balance between bone utilization and surgical simplicity. Finite element analyses have shown that the All-on-4 “M” configuration provides more favorable stress distribution along the implant axis and cortical bone compared with the standard All-on-4 layout, reducing prosthetic cantilevers and biomechanical overload [3,4]. Additionally, by tilting all four implants, clinicians can often avoid sinus augmentation or bone grafting, resulting in shorter treatment time and improved patient comfort.

Digital approaches are gathering momentum in modern dentistry. For example, a digital technique has recently been proposed for mounting a virtual articulator based on a scan of a facebow worn by the patient [27]. In the present case study, the use of a digital workflow, from virtual planning to implementation, further enhances predictability and reduces intraoperative adjustments, aligning with the clinical benefits reported by Soto-Peñaloza et al. [1].

Despite the promising results, this study has several limitations. First, it describes a single clinical case; therefore, its conclusions should be interpreted with caution. Second, bone temperature measurements, using thermocouples or infrared cameras, were not performed, so conclusions about intraosseous temperature control are based on indirect clinical indicators and in vitro literature data. Third, the accuracy of implant placement was not characterized in terms of linear or angular errors. Instead, it was estimated qualitatively, via the overlap between the virtual plan and the postoperative intraoral scan. Fourth, the follow-up period spanned only one month. Prospective in vivo trials of implant dentistry procedures involving internally cooled guides, conventional guides, and free-hand protocols could provide a statistically validated ranking of their ability to prevent bone overheating and ensure accurate implant insertion. Finally, incorporating intraoperative temperature sensors and real-time irrigation flow monitoring could represent future steps toward intelligent surgical guide systems.

## 5. Conclusions

The present clinical case report demonstrates that a fully digital workflow involving surgical guides with internal coolant channels can enable accurate and safe implant placements in complex full-arch rehabilitations.

The protocol presented in this case report ensured effective thermal control, maintaining intraosseous temperatures below the critical threshold, as indicated indirectly by the high primary stability (ISQ > 70), which allowed for immediate maxillary loading and predictable osseointegration. The flapless guided approach minimized postoperative morbidity and preserved soft-tissue architecture, contributing to rapid healing and high patient satisfaction.

Taken together, the results of this study indicate that internally cooled guided surgery may represent a feasible approach to combining the accuracy of digital planning with enhanced thermal regulation, offering a reliable, minimally invasive, and ergonomic solution for All-on-4 “M” rehabilitations.

Future clinical trials with large sample sizes and long-term follow-up are warranted to confirm the findings of this study and optimize the design of next-generation guides for dental implant placement.

## Data Availability

All the scientific data produced during this study are included in the article. The patient’s personal data and raw clinical test results are unavailable due to privacy and ethical restrictions.

## Abbreviations

The following abbreviations are used in this manuscript:

All-on-4/AO4	Full-arch oral rehabilitation concept using four dental implants
“M” configuration	Implant configuration with all four implants tilted at ~30°, forming an M-shaped geometry
CBCT	Cone Beam Computed Tomography
STL	Standard Tessellation Language or Stereolithography
CAD/CAM	Computer-Aided Design/Computer-Aided Manufacturing
PMMA	Polymethyl Methacrylate
MUA	Multi-Unit Abutment
ISQ	Implant Stability Quotient
3D	Three-Dimensional
FP1	Fixed Prosthesis Type 1
rpm	Rotations per Minute
